# Epidemiology of JIA

**DOI:** 10.1186/1546-0096-12-S1-I15

**Published:** 2014-09-17

**Authors:** Pekka Lahdenne

**Affiliations:** 1 Department of Pediatrics, Helsinki University Central Hospital, Helsinki, Finland

## 

Comparison of epidemiological studies of JIA is challenging due to many influencing factors, mainly differences in classification criteria or study designs (e.g. population-based, hospital-based, questionnaires, registry data). Recently, longitudinal cohort studies have provided important contributions to the understanding of the disease course of JIA in the long run. Latest advances in research of incidence and prevalence of JIA will be highlighted and discussed to facilitate understanding the consequences of epidemiological knowledge of the disease.

## Disclosure of interest

P. Lahdenne Speakers Bureau of: Consultancy and speaker’s fees from Pfizer, Abbvie, Roche, Novartis

